# A database of high-density surface electromyogram signals comprising 65 isometric hand gestures

**DOI:** 10.1038/s41597-021-00843-9

**Published:** 2021-02-18

**Authors:** Nebojša Malešević, Alexander Olsson, Paulina Sager, Elin Andersson, Christian Cipriani, Marco Controzzi, Anders Björkman, Christian Antfolk

**Affiliations:** 1 Department of Biomedical Engineering, Faculty of Engineering, Lund University, Lund, Sweden; 2 The BioRobotics Institute, Scuola Superiore Sant’Anna, Pisa, Italy; 3 Department of Excellence in Robotics and AI, Scuola Superiore Sant’Anna, Pisa, Italy; 4 Department of Hand Surgery, Clinical Sciences, Sahlgrenska Academy, University of Gothenburg and Sahlgrenska University Hospital, Gothenburg, Sweden

**Keywords:** Neurology, Electromyography - EMG, Biomedical engineering

## Abstract

Control of contemporary, multi-joint prosthetic hands is commonly realized by using electromyographic signals from the muscles remaining after amputation at the forearm level. Although this principle is trying to imitate the natural control structure where muscles control the joints of the hand, in practice, myoelectric control provides only basic hand functions to an amputee using a dexterous prosthesis. This study aims to provide an annotated database of high-density surface electromyographic signals to aid the efforts of designing robust and versatile electromyographic control interfaces for prosthetic hands. The electromyographic signals were recorded using 128 channels within two electrode grids positioned on the forearms of 20 able-bodied volunteers. The participants performed 65 different hand gestures in an isometric manner. The hand movements were strictly timed using an automated recording protocol which also synchronously recorded the electromyographic signals and hand joint forces. To assess the quality of the recorded signals several quantitative assessments were performed, such as frequency content analysis, channel crosstalk, and the detection of poor skin-electrode contacts.

## Abstract (summary)

**Table:** 

Measurement(s)	muscle electrophysiology trait
Technology Type(s)	electromyography • multi-electrode array system • strain gauges
Factor Type(s)	isometric hand gesture • electromyography
Sample Characteristic - Organism	Homo sapiens

Machine-accessible metadata file describing the reported data: 10.6084/m9.figshare.13625828

## Background & Summary

The electromyographic signal (EMG) encodes information related to the recruitment patterns of motor neurons innervating skeletal muscles close to the site of signal acquisition. A good understanding of the underlying electrophysiology is important for studying human biomechanics^1^ and for diagnosing neuromuscular disease^2^. Furthermore, the knowledge of the underlying neurophysiology behind motor control acquired non-invasively by surface EMG (sEMG) and high density surface EMG (HD-sEMG) has attracted increasing interest in the pursuit of novel human-computer interfaces^3^. Among salient uses for this application of the technique in the field of upper-limb prosthetics, where sufficiently accurate mappings from measured forearm myoelectricity to hand- and wrist kinematics could be used to deliver intuitive motor commands to a robotic replacement limb. At present, commercially available myoelectric prostheses are most commonly controlled via direct, proportional control of a single degree of freedom (DoF)^4^. Advanced multifunctional prostheses^5^ are within this framework typically controlled sequentially by employing some protocol for switching between active DoFs^6^. Although simple, this type of interface is often perceived as slow and unintuitive by the user, requiring nontrivial cognitive efforts and leading to a high number of users abandoning their prosthesis^7^. Efforts to improve the ability to automatically decode forearm sEMG into natural movement commands, therefore, have the potential to be of considerable value for transradial (forearm) amputees.

Despite having been the subject of studies for several decades, the exact relationship connecting sEMG to limb kinematics remains in part elusive. Due to the apparent stochasticity, nonstationarity, and nonlinearity of sEMG with respect to muscle contractions^8–10^, many studies aimed at the extraction of motor intent have found success by refraining from modelling this relationship explicitly and instead resorting to a combination of manual *feature engineering* and *machine learning*^11^. With this strategy, the information density of acquired sEMG signals is increased by compression into a set of numeric descriptors (i.e. features) via a sliding time window technique. Given that the selected features capture discriminative properties of the latent generative process, pattern recognition algorithms^12–15^ can thereafter be deployed to map such features to grasps and motions. More recently, the application of automated *feature learning* in the form of deep neural networks^16–23^ has enabled end-to-end mappings directly, from raw EMG to movement representations. Such methods are arguably uniquely appropriate for processing HD-sEMG due to the signal’s structural resemblance to conventional image data. Both feature engineering and deep learning circumvent the complexity of explicitly modelling the underlying electrophysiology, but at the cost of requiring labelled data sets containing (I) recorded EMG and (II) numeric representation of synchronous performed movements.

Multiple databases^18,24–29^ exist which contain collections of EMG or HD-sEMG together with synchronous movement stimuli and/or joint angle time series. The general importance of publicly available resources of this kind can be understood as twofold: firstly, it allows for the development of novel methods by other researchers without requiring time-consuming data collection. Secondly, it allows for inter-method collation, as the performances of different methods are difficult to compare fairly if evaluated on data sets with differing characteristics.

The aim of this data set was to contribute to the development of better myoelectric decoding schemes by presenting a new HD-sEMG data set, distinguished by a different approach compared to previous contributions to the same end. 128 channels of sEMG data were recorded at the level of the forearm from 20 able-bodied and healthy participants with a recording protocol constituted by 65 unique movements. These 65 movements were furthermore interpreted as compounds of 16 basis movements that capture the major DoFs of the hand and wrist. It is our hope that this type of decomposition will allow for multi-label machine learning^20^ approaches to be leveraged and potentially lead to the development of more dextrous control interfaces. Furthermore, forces exerted at the level of the wrist and the digits were collected and are provided here to allow for regression-type approaches, which might offer new possibilities in the domain of proportional control for prosthetic hands.

## Methods

### Participants

Twenty able-bodied volunteers (14 men and 6 women) aged between 25 and 57 years (mean age 35 years) participated in the study. All participants were right-handed and neurologically intact. All participants provided informed consent, and the study was approved by the Regional Ethical Review Board in Lund, Sweden (Dnr 2017-297).

### High density sEMG recording

The EMG signals were recorded using a Quattrocento (OT Bioelettronica, Torino, Italia) biomedical amplifier system. The Quattrocento is able to acquire up to 400 channels sampled with 16-bit resolution. In this study, the EMG recording chain comprised high density sEMG electrodes, preamplifiers with 5x gain located at the electrode connectors, amplifiers within the Quattrocento device, and the A/D converters. In total, including preamplifiers and amplifiers, the HD-sEMG signals were amplified 150 times. The EMG signals were sampled at 2048 Hz and a hardware high-pass filter at 10 Hz and a low-pass filter at 900 Hz were used during recordings.

**Fig. 1:**
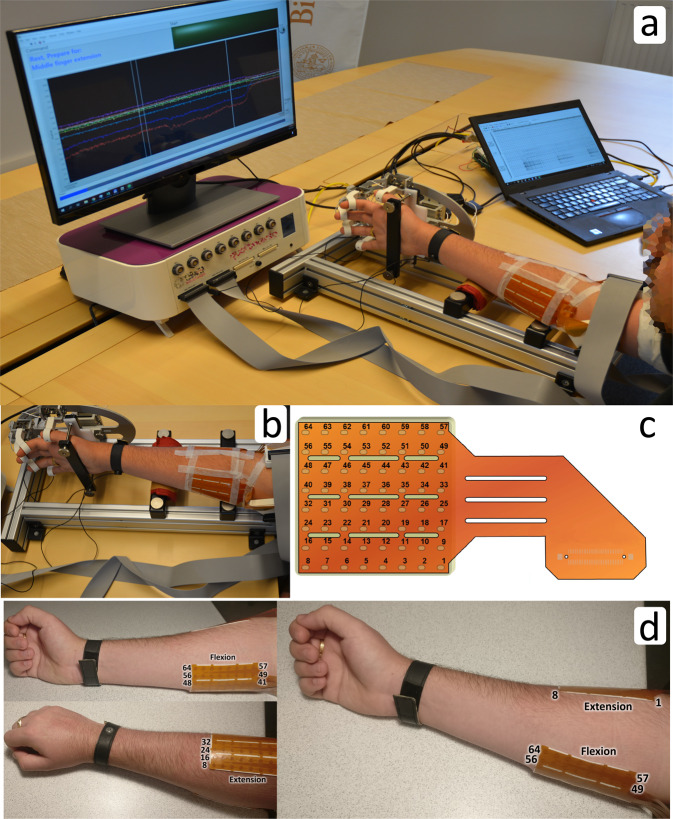
Measurement setup. (**a**) The participant’s hand was positioned inside the force measurement device. Two 64 contacts electrodes were placed on dorsal and volar aspects of the upper forearm. Preamplifiers were placed at the electrode connector and amplified EMG signals were routed to Quattrocento device. HD-sEMG signals were displayed on a laptop screen in real-time while hand forces and cues were shown on a separate screen in front of the participant. (**b**) Hand positioned inside force measurement device taken from a different angle. (**c**) Pinout of ELSCH064NM3 electrode (skin top view). The differentiation of the EMG channels was done along the increasing channel indexes. For example, the first output (ch1) is calculated as the difference between EMG signals at contacts 2 and 1, ch8 as 9-8, and the last output (ch64) as the difference between the first of the next electrode and the last of the current electrode. (**d**) Position of flexion and extension electrodes in supinated and fully pronated forearm orientations.

The electrodes used in his study consisted of 64 contacts arranged in an 8 × 8 matrix, with an inter-electrode distance of 10 mm (ELSCH064NM3, OT Bioelettronica, Torino, Italy). To reduce common-mode noise in the EMG signal, the recording was performed in a differential manner. In this mode, consecutive channels were subtracted, where the enumeration of the electrode channels is shown in Fig. 1c. Due to the electrode orientation with respect to the underlying muscles, the differentiation of the EMG signals was done along the muscle fibers. As the result, ch1 signal was calculated as the difference between EMG signals at electrode contacts 2 and 1, ch2 as the difference between signals at contacts 3 and 2, and so on. This methodology also implies that channels that are a multiple of 8 (ch8, ch16…) have different EMG signal pick-up area as contacts 8 and 9 (contacts 16 and 17, and so on) span along the whole length of the electrode. Additionally, the last channel of the electrode (ch64) is calculated as the difference between EMG signals at first contact of the next electrode (contact 1) and the last electrode contact (contact 64) of the current electrode.

Two HD-sEMG electrodes were positioned on the dorsal and the volar aspects of the forearm with the intention to cover, or partially cover, the main fingers flexors and extensors (flexor digitorum profundus – responsible for flexion of fingers D2-D5, extensor digitorum communis – responsible for extension of fingers D2-D5), wrist flexor/extensor (flexor carpi radialis, flexor carpi ulnaris – responsible for wrist flexion, extensor carpi radialis longus, extensor carpi ulnaris – responsible for wrist extension) and pronator/supinator (pronator teres, supinator), and thumb flexor/extensor (flexor pollicis longus – responsible for thumb flexion, extensor pollicis longus – responsible for thumb extension) and thumb abduction (abductor pollicis longus). As the HD-sEMG electrodes can cover a relatively large area, the positioning of the electrodes was guided by physiological landmarks, such as distance from the elbow for the distal placement, and distance from the ulna for radial orientation. The electrodes were placed approximately 3 cm from elbow (elbow to closest electrode corner) and 2 cm from ulna (edge of the ulna to edge of the electrode). The positions of the electrodes are shown in Fig. 1. An electrode consists of a thin, flexible substrate layer on which a foamy single-use double-adhesive layer is applied. The foamy layer has holes punched at the locations of electrode contacts which were filled with a conductive gel. This structure permits a tight and comfortable fit on a forearm regardless of the circumference. In addition, to ensure firm electrode contact throughout a long measurement (lasting approximately 1 h) an elastic bandage was placed over both electrodes. The reference electrode, which was in the form of a ribbon, was placed around the wrist.

The HD-sEMG signals were recorded with the OT Biolab program (OT Bioelettronica, Torino, Italia) that saves the uncompressed data in a proprietary file format.

### Isometric force recording

A custom-made force measurement device was used to obtain hand forces during the recording protocol^30^. The device was designed in a manner that enables independent acquisition of finger and wrist isometric forces. The motivation for choosing an isometric setup was to simulate muscle behaviour in a forearm amputee where the remaining muscles have a relatively small contraction amplitude. The hand position inside the force measurement device is shown in Fig. 1.

The device comprises nine strain gauges, four measuring D2-D5 flexion/extension forces, two measuring thumb flexion/extension and abduction/adduction, and three measuring wrist flexion/extension, pronation/supination, and radial/ulnar deviation. Force gauges and hand joints were interfaced through 3D printed finger braces which were specifically chosen for each participant. Using the braces, the fingers were placed in a neutral position, approximately in the middle of the range of motion. During the recording protocol, the force measurement device was placed on the table with the participant sitting in front of it. The chair height was adjusted to provide the participant with a comfortable body posture during the recordings. Although the device could be adjusted for both, right and left hand, for simplicity in this study it was used only in the right-hand setup (as all participants were right-handed).

The measured hand forces were provided as analog signals in the range of 0–5 V for the force range ± 100 N, where in neutral position (0 N) the force sensors value was 2.5 V. Within this range, the sensor output was proportional to the force with less than 1% full-scale error. The signals from the force sensors were digitalized using a NI-USB 6218 (National Instruments, Austin, Texas, USA) A/D with 16-bit amplitude resolution and sampling frequency of 200 Hz. The visualization and the recording of the signals were managed by a custom-made LabVIEW (National Instruments, Austin, Texas, USA) program. The same program controlled synchronization between the Quattrocento and the force signals by generating TTL pulses recorded by both devices. The pulses generated by one of the digital outputs of NI-USB 6218 were 0.2 s wide and occurring every 2 s. The forces presented in this paper are given in volts [V], and the transfer function between sensor analog output and the force is the following:

force = analog_voltage*40–100 [N]

The detailed description of the measurement device and its error validation could be found in our previous publication^30^.

### Recording protocol

The recording session was initiated by a brief explanation of the protocol to the participant, after which he/she signed the informed consent. Next, the chair height and finger braces were adjusted to fit the participant to ensure comfortable body posture and hand fit inside the force measurement device. Before applying the electrodes, the participant was informed about the specific hand movements to be performed during recordings. The list of all the movements was provided to the participant, and a time period was given to the participant to go through the list and try to execute the hand movements with the hand outside the force measurement device. In the case the participant felt that a specific hand movement was difficult to execute, the participant had an opportunity to ask for an explanation and practice the movement in both an isometric manner and in a free-hand manner. Upon confirming that the participant was able to perform the hand movements from the list, the electrodes were placed on the upper forearm. Subsequently, the participant was asked to place the hand into the force measurement device and perform random hand movements so that the electrode-skin contact could be assessed. At this stage, it was checked if there were any EMG channels containing high noise or spikes that occurred during the muscle contractions. The presence of a high amplitude signal was usually an indicator that some of the electrodes have poor contact with the skin surface. In these cases, the elastic bandage covering the electrodes was tightened. If there were still channels with high amplitude noise, the electrode was removed and reapplied at the same position (with a new foamy layer and gel). Upon confirming that the EMG channels were not contaminated with excessive noise nor movement artefacts the automated measurement protocol was started.

The recording protocol consisted of 66 hand movements (65 unique movements and one repeated twice) with five repetitions separated with rest periods each lasting 5 s. As the main aim of this database was to provide useful data for EMG classification, specifically for multi-label classification, the selection of movements was made so that it comprised all single degree of freedom movements (1DoF) that could be obtained by the means of the force measurement device, such as flexion/extension of individual fingers, but also, compound movements comprising combinations of basic movements. In this study, 16 basic/1DoF movements were selected, two per D2-D5 fingers (flexion-extension), four for thumb (flexion-extension-abduction-adduction) and four for wrist (flexion-extension-pronation-supination). To make the movement commands more understandable for the participants, terms flexion-extension for D2-D5 fingers were replaced with bend-stretch, flexion-extension-abduction-adduction for thumb with down-up-left-right, and flexion-extension-pronation-supination for wrist with bend-stretch-rotate anti clockwise-rotate clockwise. With 16 basic movements, the number of all the possible combinations is very high, and it would be impractical to record all of them. Thus, the subset of compound movements was derived using several rules:

1. All co-contractions of a single joint were excluded from the list. For example, a command to simultaneously flex and extend a finger was not included as the net finger force would be zero, thus it would not be detected by the force sensor.
2. For movements comprising two fingers, only flexions of adjacent fingers were included in the list. Besides being more common in activities of daily living, the flexions of adjacent fingers are movements that could be performed without specific motor skills or extensive training (unlike flexion of non-adjacent fingers).
3. Hand movements including combinations of joints flexion and extension were excluded from the list, with the exception of wrist extension that was included with fingers flexions, and pointing movement that included extension of index finger together with flexion of D3-D5 fingers. Similar to the rationale provided for rule 2, hand movements comprising a mixture of joints flexions and extensions are rare in activities of daily living, and difficult to execute.
4. For the multi-joint hand movements (>2 DoF movements), the selection was based on the most common hand grips and gestures. These movements include known muscle synergies that enable easy and intuitive execution even in an isometric fashion.
5. As the fingers extensions were underrepresented in the recording set, two instances of D2-D5 fingers extensions were included in the list of movements (movement codes 58 and 60 in Online-only Table 1).

During the pilot measurements (not included in this study), the list of movements was modified and some of the hand movements were removed if there were difficulties in performing them. In total, 66 hand movements (65 unique) were used in this protocol, see Online-only Table 1.

The measurement was guided automatically by the custom-made software developed in LabVIEW. The graphical interface presented to the participant textual commands for the next/current hand movement (as in Online-only Table 1). The onset of the hand movement was directed by a large green light indicator (visual cue) and a short beeping sound (auditory cue). The participant was supposed to “hold” the movement at a comfortable force level until the visual indicator was turned off. The movement end was also signalled by the change of the displayed textual command, which during the rest periods comprised words “Rest, prepare for: (movement from Online-only Table 1)”. Each hand movement was performed five times before switching to the next movement. The transition between different movements was additionally highlighted by the change of the text colour, which toggled between red and blue after the fifth repetition of a movement. This feature was added as it was noted during the pilot recordings that the participants tend to focus more on the movement onset cue than on the textual command. This often resulted in producing the same hand movement for extra repetitions although the movement textual command was changed. With the colour change between different hand movements, the occurrence of wrong repetitions was significantly reduced.

### Data processing

The HD-sEMG data recorded with OT Biolab was filtered offline to further remove power line noise. A zero-phase 3^rd^ order band-pass Butterworth filter centered at 50 Hz with 4 Hz width implemented in Matlab (command: *filtfilt*) was used for this task. No additional processing was done to the HD-sEMG signals.

The signals acquired with two devices (Quattrocento and NI-USB 6218) and recorded with two programs running in parallel (OT Biolab and LabVIEW) were synchronized using the common TTL pulses that were supplied to both recording chains. In the offline processing, these pulses were detected in both files and the signals were truncated so that the beginning was at the leading edge of the first pulse, while the end was at the trailing edge of the last pulse. As an additional check, the pulses were counted in both files to verify that there was no missing data. To join the HD-sEMG data sampled at 2048 Hz and force and movement label data sampled at 200 Hz, interpolations were performed so that all the signals matched the HD-sEMG sampling rate (2048 Hz). This step was done using Matlab command *interp1*.

**Fig. 2:**
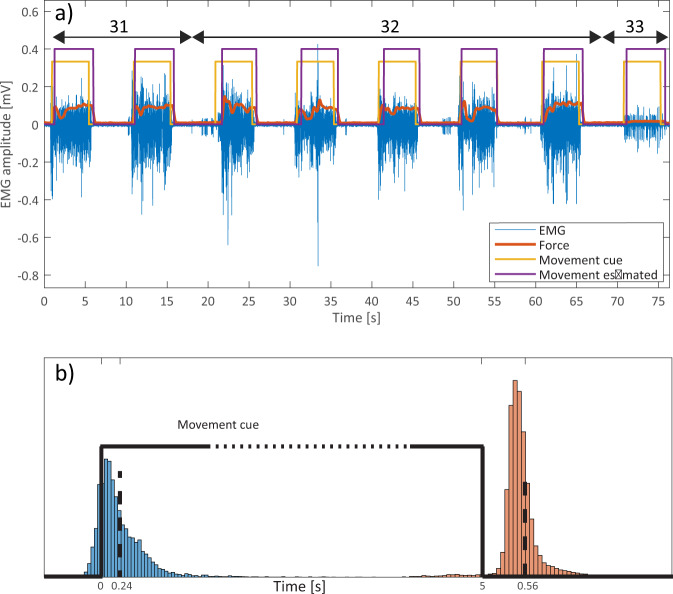
An example of the recorded signals, markings, and histograms of latencies for movement onsets and cessations. (**a**) shows an EMG channel (out of 128) from the electrode placed over the volar portion of the forearm. Superimposed with the EMG signal (blue) are ring finger force (red), movement cue timings presented to the participant (yellow), and the re-labelled movement durations estimated using force levels (purple). This signal example comprised movements 31 (Ring finger: bend + Wrist: rotate anti-clockwise), 32 (Ring finger: bend + Wrist: rotate clockwise) and 33 (Middle finger: bend + Index finger: bend). From the figure, it could be noted how the force fluctuates during movements, similarities, and differences between consecutive repetitions of the same movement, but also clear differences between the EMG and the force profiles when switching to a completely different finger (joint). (**b**) shows latencies of re-labelled movements for all repetitions, movements, and participants. Mean latencies for onset was 0.24 s, while for the cessation was 0.7 s.

The onset and cessation of each movement were presented to the participant using visual and audio cues. Nevertheless, the hand movement was usually delayed due to physiological reaction time, but also due to reduced focus of the participant during prolonged measurement. Thus, the real movement onset and cessation were not matching the movement labels that correspond to movement cues. To provide movement labels that correspond to real hand movements, a temporal re-labelling was done (see Fig. 2a). The main aim of this process was to provide movement (class) labels that separate rest periods from movements, thus providing consistent signals for training procedure of a classifier, but also providing better segmented data for classifier testing purposes. The temporal re-labeling relied on measured hand forces as the indicators of the movement offset and cessation. The algorithm of temporal re-labeling can be described as the sequence of the following steps:

- In the period preceding the movement cue, the “rest” hand forces were obtained as the average values within a 1 s window. This resulted in a 1 × 9 vectors (one for each sensor) that was updated before each movement cue.
- The “rest” hand forces were subtracted from each force channel. This process was done in a loop that stepped through the signal in a sample-by-sample manner, where the “rest” vector was updated at the beginning of each movement cue.
- Subtracted signals were rectified and all 9 force channels were summed together for each sample, resulting in a 1 x (signal_length) vector.
- For each hand movement, the algorithm found the minimal hand force within 1 s period preceding the movement onset cue, and maximal hand force during 5 s after the movement onset cue.
- The real movement onset was then defined as the sample at which the summed force signal was above 50% of the difference between minimal and maximal movement forces.
- A similar procedure was done for estimating when the real hand movement ended. For each hand movement, the algorithm found the maximal hand force within 1 s period preceding the movement cessation cue, and minimal hand force during the 5 s after the movement cessation cue.
- The real movement cessation was then defined as the sample at which the summed force signal crosses below 50% of the difference between minimal and maximal movement forces.

Calculated latencies between the presented cue and the real movement were joined for all participants, movements, and repetitions (Fig. 2b). Based on the cumulative data, mean latencies were extracted; 0.24 s (std = 0.33 s) for onset and 0.56 s (std = 0.46 s) for cessation. It should be noted that movement onset was expressed by both auditory and visual cue, while movement cessation was only expressed by turning off of the visual cue (onscreen virtual LED). This fact could be used to explain the differences between latency distributions for onset and cessation of the movements. Another difference between these two conditions is that for onset, participants were focusing on the incoming cues, while for the movement cessations, the participants were focusing on maintaining the desired hand gesture, and transitioning to rest state required more time. Finally, as the movement executions were rhythmical (5 s on, 5 s off, 5 s on…) it was possible to anticipate the timing of the onset cue, which is also the reason for some early movement onsets (before actual cue). The observed movement latencies (relabelling results) are comparable with previous studies focused on human reaction time^31,32^.

## Data Records

The data presented in the current paper can be downloaded from figshare^33^ and used freely for any purpose. This section describes the contents of the provided files.

Each file, encoded in*.mat* format, contains data recorded from a single test participant and is named according to the convention *sx.mat*, where x is the participant index (i.e. an integer in the range 1–20). Each file contains a set of variables, listed below, together constituting the data and metadata connected to the participant. *L* here denotes the total number of sampled time points across the entirety of the recording session.

- *subject*: An integer in the range 1–20, representing the participant number (same as in file name).
- *Fs*: The sampling rate of the HD-sEMG signal and of the synchronized forces and movement stimuli signals, here always equal to 2048 Hz.
- *emg_extensors*: An *L* × 8 × 8 matrix containing the sEMG samples of the 64 channels recorded from the dorsal side of the forearm. The second and third matrix indices correspond to the relative positions of the corresponding electrodes along and across the forearm, respectively.
- *emg_flexors*: An *L* × 8 × 8 matrix containing the sEMG samples of the 64 channels recorded from the volar side of the forearm. The second and third matrix indices correspond to the relative positions of the corresponding electrodes along and across the forearm, respectively.
- *force*: A *L* × 9 matrix containing the 6 force channels, samples synchronized with those of the EMG signals.
- *class*: An 1D array of length L containing integers in the range [0,65] which encodes the class of the movement stimuli being presented to the participant concurrently with collected sEMG and forces. Array elements of value 0 denote the rest state.
- *labels*: An *L* × 16 Boolean matrix, computed directly from the *class* variable via a lookup table. The truth value of *labels*(i, j) is 1 if the movement which the participant is prompted to perform at time j incorporates the i:th DoF and 0 if it does not.
- *repetition*: An 1D array of length L containing integers in the range 0–5 which encodes the repetition number of the movement stimuli being presented to the participant.
- *adjusted_class*: The *class* variable following automated re-labelling as described in the Data Processing section.
- *adjusted_labels*: The *labels* variable following automated re-labelling as described in the Data Processing section.
- *adjusted_repetition*: The repetition variable following automated re-labelling as described in the Data Processing section.
- *outlier_scores_extensors*: An 8 × 8 matrix containing channel-specific outlier scores, computed via the method described in the Technical Validation section, of the sEMG channels presented in *emg_extensors*. The value stored in *outlier_scores_extensors[i, j]* corresponds to the channel *emg_extensors[i, j, :]*.
- *outlier_scores_flexors*: An 8 × 8 matrix containing channel-specific outlier scores, computed via the method described in the Technical Validation section, of the sEMG channels presented in *emg_flexors*. The value stored in *outlier_scores_flexors[i, j]* corresponds to the channel *emg_flexors[i, j, :]*.

## Technical Validation

In addition to the protocol followed during recording sessions for ascertaining the quality of acquired signals (described in the Methods section), the resulting dataset was subsequently assessed quantitatively in an offline setting. This validation of HD-sEMG signals entailed (i) extracting signal frequency spectrums from all EMG channels, (ii) computing the cross-correlations between all possible pairs of channels for all recording sessions, and (iii) computing a channel outlier metric separately for each participant and EMG channel. The details and implications of these approaches are given in the following sections.

### Frequency spectra

**Fig. 3:**
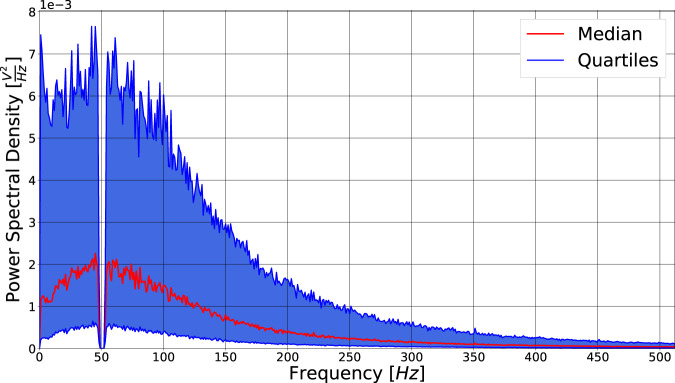
Aggregated representation of HD-sEMG signal spectra from all participants, movements, and channels. The central red line represents the median spectrum and the blue regions represent the quartiles (computed separately for each frequency bin).

The frequency spectra of the EMG signals comprising the current dataset are summarized graphically in Fig. 3. A single spectrum was computed for each participant and channel (20 128 = 2560 spectrograms in total) using Welsh’s method; the curves plotted in Fig. 3 represents quartiles, computed frequency-wise, over all such spectra. Excluding the discontinuities induced by offline notch filtering at 50 Hz power line interference, the morphologies of the spectra correspond to those expected in light of the previous studies^34^. As it could be expected, there was a notable variation in average amplitude between deciles, which is a result of the variation in average amplitude between channels – the 128 electrodes cover muscles situated at different depths, and different muscles are moreover recruited for different numbers of unique movement classes, resulting in significant variation in amplitude and by extension observable variations in vertical offsets of deciles.

### Channel correlations

**Fig. 4:**
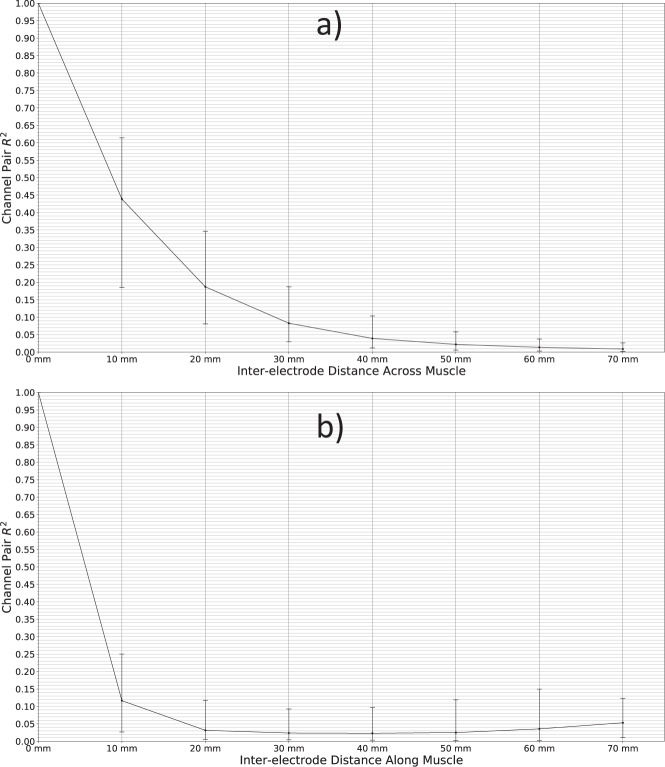
Coefficient of determination *R*^2^ of channels pairs for all 8 possible inter-electrode distances **(a)** perpendicular to muscles and **(b)** parallel to muscles. The central markers represent means and the upper and lower delimiters of the error bars represent the 75^th^ and 25^th^ percentile, respectively, computed across all participants and possible channel pairings.

The two HD-sEMG electrode arrays used for the current study were, during the experiments, placed on the skin in a way to cover multiple forearm muscles. Consequently, when a participant attempted to perform a movement, a specific subset of covered muscles was recruited, leading to a spatially clustered pattern of activity in the concurrent HD-sEMG. This behavior is the consequence of the nature of the electromyography technique that relies on the electrical signal traversing along the muscle fibers (action potential) which is then transmitted through tissues radially, eventually reaching recording electrodes. The amplitude of this signal on different electrodes is proportional to the distance between the source (muscle) and the recording points (electrodes). The zero-lag cross-correlation coefficient between a given pair of EMG channels is therefore expected to be large for channels with electrodes situated close together, and small for channels with electrodes situated far apart. In the event of non-negligible interference or other types of noise shared across multiple channels (e.g. excessive motion artefacts), this regularity can no longer be expected to hold true, as even signals acquired by electrodes far apart would exhibit notable covariation. To verify the absence of this type of noise in the presented dataset, the zero-lag cross-correlation coefficient between every possible pair of channels was computed for each participant and compared to the physical distance separating the electrodes of the pair. The analysis was performed on whole signals (comprising all movements) for all electrode pairs within the same row or column, and for all the participants. Only pairings where both channels belong to the same electrode row/column were used. This also means that only channels within the same electrode were considered, as the distance separating the two 8 × 8 electrode arrays was not noted. As the electrodes were coarsely aligned with the direction of muscle fibers of major muscles within the forearm, the electrical activity picked by electrode rows (channels 1-2-3… 63–64) and columns (channels 1-9-17-… 56–64) is resulting from different physical processes. In the case of electrode rows, the electrical signal is directly coupled with the propagation of action potentials along the muscle fibers, while in the case of electrode columns the signal is reaching recording sites by passive radial propagation from muscle fibers through surrounding tissues. To observe both of these effects the cross-correlation was calculated for the two directions separately and is presented in Fig. 4. In Fig. 4a, the observed relationship of the inter-electrode distance across muscles and channel cross-correlation is presented; Fig. 4b contains the observed relationship of the distance along muscles and channel cross-correlation. In both cases, a strong negative relationship was observed between the variables, indicating that the amount of common noise and crosstalk was limited, but it is notable that the channel cross-correlation decreases much slower in the case of electrodes placed across the muscle. As mentioned before, this behavior reflects underlying electrophysiology coupled with the zero-lag cross-correlation method which was used to assess channels crosstalk. The employed method results in (1) higher cross-correlation values between channels across the muscle where the signal from muscle fibers is only attenuated in proportion to the electrode-fiber distance (Fig. 4a), and (2) lower cross-correlation when the signal is propagated with some time delay (Fig. 4b).

### Outlier scores

As the used HD-sEMG electrodes have some mechanical constraints, such as limited curvature and adhesion properties, there is always a possibility of having a poor skin-electrode contact at specific electrode portions. This issue results in an increased environmental noise level (50 Hz) and the appearance of movement artifacts in the form of high-amplitude spikes during some specific contractions that deform the forearm surface more than the electrode can follow. Thus, the signal quality of HD-sEMG can plausibly be expected to vary across channels for a given participant and recording session. Although the movement artifacts spikes are usually very sparse, as they appear together with some specific hand gestures, they could potentially, impede some of the machine-learning algorithms by providing false EMG behavior.

In subsequent offline processing of signals, it may thus be of interest to exclude channels deemed as *outliers* by some appropriate measure of channel deviation. In order to provide such a measure in the current dataset, a simple *outlier score* was defined and calculated for each individual channel and recording session. To calculate this score, denoted *O*_*i*_ for the *i*^th^ channel, the following procedure was carried out: initially, the 99^th^ percentile of all rectified voltages sampled from each channel, denoted ${p}_{i}^{99 \% }$. for the *i*^th^ channel, was calculated. This value approximates the voltages reached by the considered channel at signal peaks. This statistical measure proved to be more robust than simply finding the maximum voltage across all samples. Next, the first and third quartile (Q1 and Q3, respectively) were extracted from the lists of all such values across all channels (${p}_{i}^{99 \% }$ for all *i*) and used to compute the interchannel *interquartile range* as $IQR=Q3-Q1$. In accordance with one common definition of a statistical outlier, a nonzero outlier score *O*_*i*_ was lastly assigned to the *i*^th^ EMG channel if and only if ${p}_{i}^{99 \% }$ exceeded a threshold voltage given by $T=Q3+1.5\cdot IQR$. For such channels, i.e. where ${p}_{i}^{99 \% } > T$, the value of *O*_*i*_ was set to be proportional to the number of IQRs with which ${p}_{i}^{99 \% }$ exceeded the threshold:

$${O}_{i}=\frac{{\rm{\max }}\left(0,\,{p}_{i}^{99{\rm{ \% }}}-T\right)}{IQR}$$

A channel with an outlier score *O*_*i*_ = 0 can be interpreted as falling within expected boundaries of valid EMG amplitude variation. For a channel with nonzero *O*_*i*_, the outlier score is intended to quantify the degree to which the channel generated notably higher peak voltages, as caused by e.g. signal high-amplitude spiking, than those of the other channels. With the provided list of scores, it is possible to exclude channels at an arbitrary level of acceptable channel deviation.

Due to the bipolar sampling protocol used to acquire EMG signals during recording sessions, an outlier score was computed only for the $128-(8\cdot 2)=114$ channels not originating from the electrodes at the proximal end of the two electrode arrays (the remaining 16 channels were automatically given an outlier score of 0). In the current database, recording sessions contained an average of 92.24% (SD 3.61%) channels with an outlier score of 0. Among channels with nonzero outlier score, the mean value of *O*_*i*_ was calculated as 2.63 (SD 3.68).

In addition, due to the bipolar setup, channel 128 (the last channel of the second electrode) should not be used as it was not referenced in the same manner as other channels.

## Data Availability

The signal recording was performed using two programs in parallel: OT BioLab version 2.0.6254 available at www.otbioelecttronica.com for recording HD-sEMG and synchronization signals, and the custom recording software developed in LabVIEW 2016 for force signals recording, generating synchronization pulses, visualizing forces, and generating commands and cues. Data post-processing was done in Matlab and Python. The custom codes for temporal re-labeling and outlier scores are available at the GitHub repository: https://github.com/Neuroengineering-LTH/HDsEMG-database-Associated-codes.

## Online-only Table

**Online-only Table 1:** List of hand movements included in this study, as presented to the participants (shown on screen).

Code	Movement	
1	Little finger: bend	**1DoF**
2	Little finger: stretch	
3	Ring finger: bend	
4	Ring finger: stretch	
5	Middle finger: bend	
6	Middle finger: stretch	
7	Index finger: bend	
8	Index finger: stretch	
9	Thumb: down	
10	Thumb: up	
11	Thumb: left	
12	Thumb: right	
13	Wrist: bend	
14	Wrist: stretch	
15	Wrist: rotate anti-clockwise	
16	Wrist: rotate clockwise	
17	Little finger: bend + Ring finger: bend	**2DoF**
18	Little finger: bend + Thumb: down	
19	Little finger: bend + Thumb: left	
20	Little finger: bend + Thumb: right	
21	Little finger: bend + Wrist: bend	
22	Little finger: bend + Wrist: stretch	
23	Little finger: bend + Wrist: rotate anti-clockwise	
24	Little finger: bend + Wrist: rotate clockwise	
25	Ring finger: bend + Middle finger: bend	
26	Ring finger: bend + Thumb: down	
27	Ring finger: bend + Thumb: left	
28	Ring finger: bend + Thumb: right	
29	Ring finger: bend + Wrist: bend	
30	Ring finger: bend + Wrist: stretch	
31	Ring finger: bend + Wrist: rotate anti-clockwise	
32	Ring finger: bend + Wrist: rotate clockwise	
33	Middle finger: bend + Index finger: bend	
34	Middle finger: bend + Thumb: down	
35	Middle finger: bend + Thumb: left	
36	Middle finger: bend + Thumb: right	
37	Middle finger: bend + Wrist: bend	
38	Middle finger: bend + Wrist: stretch	
39	Middle finger: bend + Wrist: rotate anti-clockwise	
40	Middle finger: bend + Wrist: rotate clockwise	
41	Index finger: bend + Thumb: down	
42	Index finger: bend + Thumb: left	
43	Index finger: bend + Thumb: right	
44	Index finger: bend + Wrist: bend	
45	Index finger: bend + Wrist: stretch	
46	Index finger: bend + Wrist: rotate anti-clockwise	
47	Index finger: bend + Wrist: rotate clockwise	
48	Thumb: down + Thumb: left	
49	Thumb: down + Thumb: right	
50	Thumb: down + Wrist: bend	
51	Thumb: down + Wrist: stretch	
52	Thumb: down + Wrist: rotate anti-clockwise	
53	Thumb: down + Wrist: rotate clockwise	
54	Wrist: bend + Wrist: rotate anti-clockwise	
55	Wrist: bend + Wrist: rotate clockwise	
56	Wrist: stretch + Wrist: rotate anti-clockwise	
57	Wrist: stretch + Wrist: rotate clockwise	
58	Extend all fingers (without thumb)	**sDoF**
59	All fingers: bend (without thumb)	
60	Extend all fingers (without thumb)	
61	Palmar grasp	
62	Wrist: rotate anti-clockwise with the Palmar grasp	
63	Pointing (index: stretch, all other: bend)	
64	3-digit pinch	
65	3-digit pinch with Wrist: anti-clockwise rotation	
66	Key grasp with Wrist: anti-clockwise rotation	

1DoF movements comprise individual fingers flexions and extensions, thumb flexions, extensions, abductions and adductions, and wrist flexions, extensions, pronations and supinations. 2DoF movements comprise some of the 1DoF movements combinations. sDoF movements comprise some of the most common synergistic multi-joint hand movements.
